# Effects of Dietary Bio-Fermented Selenium Supplementation on Growth, Immune Performance, and Intestinal Microflora of Chinese Mitten Crabs, *Eriocheir sinensis*

**DOI:** 10.3390/ijms25179219

**Published:** 2024-08-25

**Authors:** Zhichao Yang, Jialin Li, Yuhan Ma, Zihao Wu, Jiaming Li, Fengchi Wang, Yuting Xi, Yusheng Jiang, Shu Huang, Qilin Yi

**Affiliations:** 1 College of Aquaculture and Life Science, Dalian Ocean University, Dalian 116026, China; onz17941@163.com (Z.Y.); lijialin724@163.com (J.L.); mayuhan3@126.com (Y.M.); 18626932498@163.com (Z.W.); 17866561266@163.com (J.L.); wfc13841172169@163.com (F.W.); 15888679502@163.com (Y.X.);; 2 Dalian Key Laboratory of Breeding, Reproduction and Aquaculture of Crustaceans, Dalian 116023, China

**Keywords:** *Eriocheir sinensis*, Bio−Se, growth performance, immune response, intestinal microflora

## Abstract

Selenium is a vital trace mineral that is crucial for maintaining regular biological processes in aquatic animals. In this study, a four-week dietary trial was carried out to assess the impact of bio-fermented selenium (Bio−Se) on the growth and immune response of Chinese mitten crabs, *Eriocheir sinensis*. The crabs were randomly allocated to five dietary treatment groups, each receiving a different dose of Bio−Se. The doses included 0, 0.3, 0.6, 1.5, and 3.0 mg/kg and were accurately measured in basal diet formulations. The results showed the weight gain rate (WGR), specific growth rate (SGR), and survival rate (SR) in the 1.5 mg/kg Bio−Se group were the highest, and 3.0 mg/kg of Bio−Se has an inhibitory effect on the WGR, SGR, and SR. The activities of the immune enzymes, including glutathione peroxidase (GPX), superoxide dismutase (SOD), and acid phosphatase (ACP), of the hepatopancreas were significantly (*p* < 0.05) increased in the 1.5 mg/kg Bio−Se group, while they decreased (*p* < 0.05) in the 3.0 mg/kg feeding group compared to the 0 mg/kg feeding group. The concentration of maleic dialdehyde (MDA) exhibited the opposite pattern. Similarly, the mRNA expression levels of antimicrobial peptides (ALF-1, Crus-1, and LYS), ERK, and Relish genes were also observed to be the highest in the 1.5 mg/kg Bio−Se group compared with the other groups. Furthermore, the administration of 1.5 mg/kg of Bio−Se resulted in an increase in the thickness of the intestinal plica and mucosal layer, as well as in alterations in the intestinal microbial profile and bacterial diversity compared to the dose of 0 mg/kg of Bio−Se. Notably, the population of the beneficial bacterial phylum *Fusobacteria* was increased after crabs were fed the 1.5 mg/kg Bio−Se diet. In conclusion, the oral administration of 1.5 mg/kg of Bio−Se improved the growth efficiency, antioxidant capabilities, immunity, and intestinal health of *E. sinensis*. Through a broken-line analysis of the WGR against dietary Bio−Se levels, optimal dietary Bio−Se levels were determined to be 1.1 mg/kg. These findings contribute valuable insights to the understanding of crab cultivation and nutrition.

## 1. Introduction

Inorganic elements or minerals play key roles in physiological processes [1]. Selenium (Se) is an important component of glutathione peroxidase, which is essential for protecting the body from oxidative damage caused by free radicals [2,3,4]. Currently, Se is regarded as an essential nutrient element for aquatic animals [5]. Research has shown that Se can elevate the synthesis of growth hormone and promote growth in fish [6]. The weight gain rate and survival rate of *Pagrus major* were increased after adding 1 mg/kg of selenium nanoparticles [7]. Moreover, appropriate dietary Se has also been reported to enhance antioxidant defense in aquatic animals. The results suggest that selenomethionine supplementation could promote the activities of lysozyme, catalase, and glutathione peroxidase, ultimately leading to an enhancement in the antioxidant capacity of *Nile tilapia* [8]. Serum phenol oxidase (PO) and lysozyme activities were significantly increased after feeding with yeast Se in *Macrobrachium nipponense* [9].

However, as a trace element, Se supplementation requires a suitable additive amount. It has been demonstrated that Se deficiency can inhibit antioxidant capacity [10,11,12], whereas supercritical dietary Se has a toxic effect on immune defense in animals [13,14]. For example, excessive Se supplementation can inhibit the growth rate and significantly decrease the hematocrit value and hemoglobin concentration while up-regulating glucose and glutamic-pyruvic transaminase (GPT) levels in *P. major* [15]. Similarly, excessive dietary Se led to a significant decline in the growth rate of catfish and induced oxidative stress [16]. Furthermore, research has elucidated that elevated Se concentration administration can markedly affect the locomotive dynamics and tactile acuity in embryonic *Danio rerio* [17]. Skeletal deformities of the spine and head of the fish were demonstrated to be associated with excess Se in aquatic environments [18]. Consequently, determining the optimal dietary Se concentration for aquatic animals is essential.

At present, the common forms of selenium in nature are organic selenium and inorganic selenium [19], and usually, organic selenium is more easily absorbed compared to inorganic selenium [20]. Biological selenium (Bio−Se) that is cultivated by lactic acid bacteria belongs to new bioavailable sources of organic selenium and has a similar function to natural selenium [21]. Until now, Bio−Se has been widely applied in aquatic animals. For instance, the growth rate and activities of immune enzymes such as glutathione peroxidase (GPX), superoxide dismutase (SOD), and acid phosphatase (ACP) were significantly increased in *D. rerio* fed with a Bio−Se diet [22]. Furthermore, it has been discovered that an excessive amount of Bio−Se is likewise toxic. Sea cucumber fed with excessive Bio−Se exhibited a significant decrease in body weight protein efficiency ratio [23]. The excessive Bio−Se diet can cause oxidative stress by decreasing SOD in fish [22]. Research has indicated that the optimum concentration of Bio−Se varied across various aquatic animals (Table 1). For example, studies have shown that the recommended dose of Bio−Se for fish species such as *Piaractus mesopotamicus*, *Oncorhynchus mykiss*, *Ctenopharyngodon idellus,* and *Argyrosomus regius* is 1.15 mg/kg [3], 3.53 mg/kg [24], 0.92–1.03 mg/kg [25], and 3.98 mg/kg [26], respectively, while in crustaceans, values of 0.40 mg/kg, 1.07 mg/kg, 0.40–0.60 mg/kg, and 0.20 g/kg were recommended as the optimal Bio−Se contents for *Litopenaeus vannamei* [27], *M. nipponense* [9], *Eriocheir sinensis* [28], and *Cherax cainii* [29]. In addition, the appropriate amount of Bio−Se for *Apostichopus japonicus* was found to be 0.50–1.00 mg/kg [8], whereas for *Haliotis discus hannai*, it was 0.32–0.33 mg/kg [30].

The Chinese mitten crab, *E. sinensis*, one of the important crustacean species with high economic and nutritional value, is extensively cultivated in China [31,32]. However, the deficiency of trace elements can lead to the weakening of immune defense in crabs [33,34], thus rendering them susceptible to pathogen invasion under high-density culture conditions [35]. In recent years, formula feed has gradually replaced fish meal and conventional dietary regimens, making it the primary source of feed for the *E. sinensis* aquaculture industry [36], while the reduction in fish meal content in aquatic feed formulations decreased the positive effect of trace elements, which ultimately affected the health and growth of the cultivated aquatic species [37,38]. Furthermore, in intensive aquaculture, crustaceans are unlikely to obtain enough Se from the environment; therefore, dietary Se has become an important aspect of aquaculture practices [39]. Some studies have reported the effects of dietary Se supplementation on crustacean growth and immunity. For example, it was reported that an appropriate Se diet can promote the growth and antioxidant capacity of *M. nipponense* [9]. The shrimp fed a diet supplemented with 0.45 mg/kg of selenium exhibited a higher weight gain rate in comparison to those fed with either 0.13 or 0.20 mg/kg of Se [39]. Feeding with selenium-enriched corn augments enhanced the antioxidant capacity of *E. sinensis* [40]. Furthermore, adding nano-Se to the diet of crabs markedly elevated the antioxidant defenses under hypoxic conditions and extended their capacity to respond to hypoxic stress [41]. However, to our knowledge, there are few reports about the effects of dietary Bio−Se supplementation on the growth and immunity of *E. sinensis*. In this investigation, different concentrations of Bio−Se were added to a basal diet formulation to explore the effects of different Bio−Se levels on the growth, immune performance, and intestinal microflora of *E. sinensis*. This study aimed to characterize the regulatory role of Bio−Se on the growth and immunity of crabs, thus establishing reference data for the efficient utilization of dietary Bio−Se for aquatic crustaceans.

## 2. Results

### 2.1. Growth Performance

**Figure 1:**
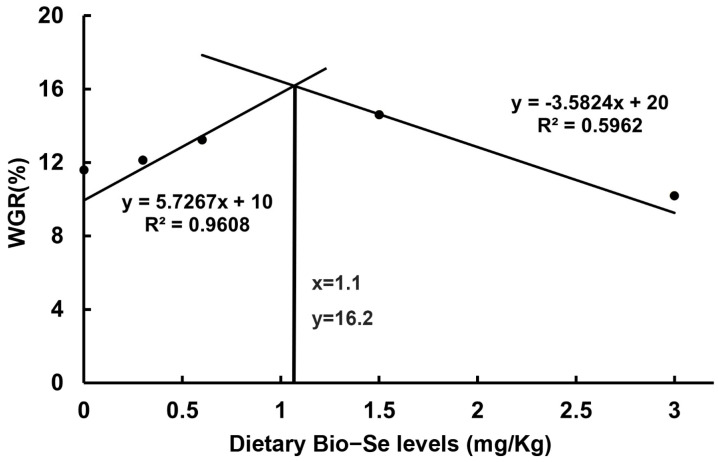
Relationship between weight gain rate (WGR) and dietary Bio−Se levels based on a broken-line regression analysis.

After a 4-week trial, the carbs fed with 0.3 mg/kg, 0.6 mg/kg, and 1.5 mg/kg of Bio−Se diet showed a significant increase in weight gain rate (WGR) compared to those in the 0 mg/kg and 3.0 mg/kg Bio−Se groups (*p* < 0.05). Similarly, the specific growth rate (SGR) in the 1.5 mg/kg Bio−Se group was significantly (*p* < 0.05) higher than that in other groups. The weight gain rate was highest in the 1.5 mg/kg Bio−Se group and lowest in the 3.0 mg/kg Bio−Se group. Meanwhile, the survival rate (SR) of crabs in Bio−Se diets (0.3, 0.6, and 1.5 mg/kg) were also higher (*p* < 0.05) than that in the 0 mg/kg and 3.0 mg/kg Bio−Se group, with the highest rate in the 1.5 mg/kg Bio−Se group (Table 1). Furthermore, a broken-line analysis of WGR against dietary Bio−Se levels was conducted, which indicated that the optimal supplementation dose of Bio−Se level was estimated to be 1.1 mg/kg diet (Figure 1).

### 2.2. Activities of Antioxidant Enzyme

**Figure 2:**
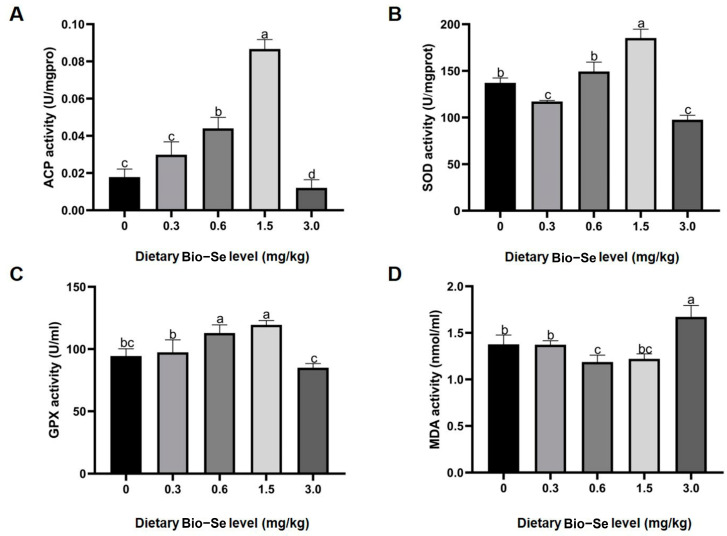
Effects of different levels of dietary Bio−Se on activities of (**A**) acid phosphatase (ACP), (**B**) superoxide dismutase (SOD), (**C**) glutathione peroxidase (GPX), and (**D**) maleic dialdehyde (MDA) in hepatopancreas. The data are shown as mean ± standard deviation (*n* = 3). Different letters indicate significant differences at *p* < 0.05 and no significant differences with the same letters (*p* > 0.05).

The activities of acid phosphatase (ACP) and glutathione peroxidase (GPX) exhibited similar expression patterns, which were up-regulated with the increasing Bio−Se concentration (0.3, 0.6, and 1.5 mg/kg) compared to the groups treated with 0 mg/kg and 3.0 mg/kg of Bio−Se. Furthermore, the level of superoxide dismutase (SOD) activity in the 0.6 mg/kg and 1.5 mg/kg Bio−Se feeding groups was similarly higher than that in the 0 mg/kg, 0.3 mg/kg, and 3.0 mg/kg groups. The ACP, SOD, and GPX activities were highest in the 1.5 mg/kg Bio−Se group and decreased to a minimal level in the 3.0 mg/kg Bio−Se group (Figure 2A–C). In contrast, the maleic dialdehyde (MDA) concentration was lower in 0.6 mg/kg Bio−Se and 1.5 mg/kg Bio−Se group, while it was highest in the 3.0 mg/kg Bio−Se group (Figure 2D).

### 2.3. Immunity-Related Genes Expression

**Figure 3:**
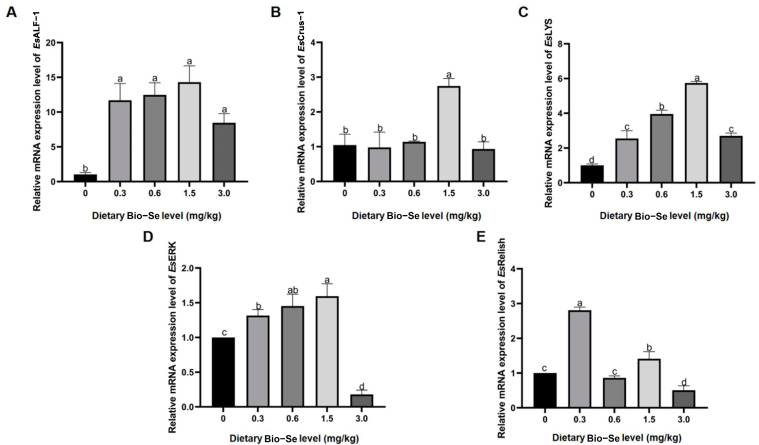
The mRNA expression levels of (**A**) *Es*ALF-1, (**B**) *Es*Crus-1, (**C**) *Es*LYS, (**D**) *Es*ERK, and (**E**) *Es*Relish in hepatopancreas at different levels of dietary Bio−Se determined by qRT-PCR. Data are shown as mean ± standard deviation (*n* = 3). *Es*ALF-1, anti-lipopolysaccharide factors-1; *Es*Crus-1, Crustin-1; *Es*LYS, Lysozymes; *Es*ERK, extracellular signal-regulated kinase. Different letters are significantly different at *p* < 0.05.

Based on the results, the expression of antimicrobial peptides (AMPs), including *Es*ALF-1, *Es*Crus-1, and *Es*LYS, was elevated with increasing dietary Bio−Se concentrations, with the highest levels in the 1.5 mg/kg Bio−Se group. The expression levels of *Es*ALF-1, *Es*Crus-1, and *Es*LYS in the 1.5 mg/kg Bio−Se group were 11.7-, 12.5-, and 14.3-fold of that in the 0 mg/kg Bio−Se group (*p* < 0.05) (Figure 3A–C). The AMP expression increase fold in the 3.0 mg/kg Bio−Se group related to the 0 mg/kg Bio−Se group was lower than that of 0.3 mg/kg, 0.6 mg/kg, and 1.5 mg/kg Bio−Se groups compared to the 0 mg/kg Bio−Se group (Figure 3A–C).

Similarly, the transcripts of *Es*ERK showed a significant (*p* < 0.05) up-regulation following the increasing Bio−Se concentration (0.3 mg/kg, 0.6 mg/kg, and 1.5 mg/kg), with the highest level in the 1.5 mg/kg Bio−Se group. Meanwhile, 3.0 mg/kg of Bio−Se significantly (*p* < 0.05) inhibited the expression level of *Es*ERK (Figure 3D). For the expression pattern of *Es*Relish, it was found that the expression was increased in the 0.3 mg/kg and 1.5 mg/kg Bio−Se group and decreased in the 3.0 mg/kg Bio−Se group compared to that in the 0 mg/kg group (Figure 3E).

### 2.4. Intestinal Morphology

**Figure 4:**
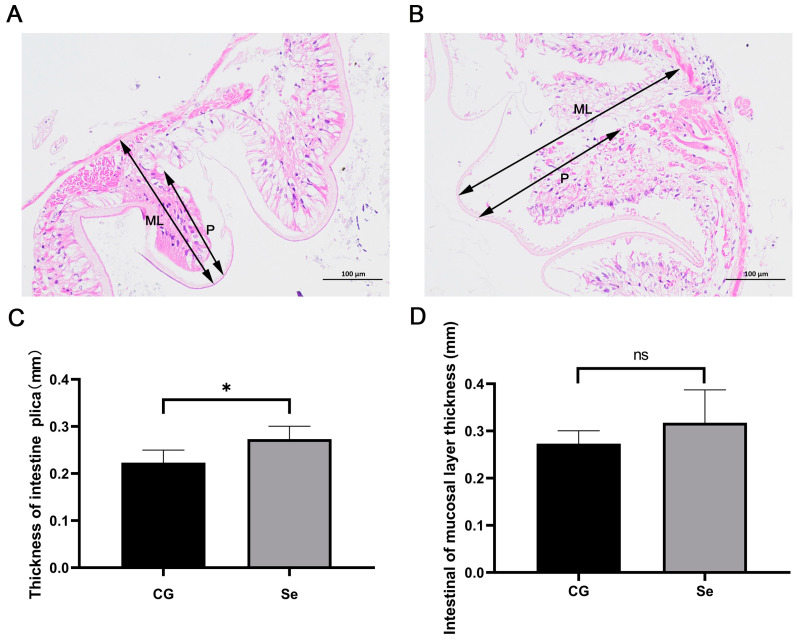
The intestinal morphology of *E. sinensis* in the control group and Bio−Se group. The intestinal morphology determined by HE staining in control group (**A**) and Bio−Se group (**B**). The statistical analysis of the thickness of intestine (**C**) and thickness of intestine mucosal layer (**D**). P, the thickness of intestinal plica; ML, the thickness of intestinal mucosal layer; CG, control group (0 mg/kg of Bio−Se); Bio−Se, Bio−Se group (1.5 mg/kg of Bio−Se); data are shown as mean ± standard deviation (*n* = 3); * *p* < 0.05; ns, no significant difference.

The intestinal morphology of the *E. sinensis* in the control group (0 mg/kg of Bio−Se) and Bio−Se group (1.5 mg/kg of Bio−Se) were observed by HE staining (Figure 4A,B). Based on the HE staining of intestine sections, greater thickness of intestinal plica (P) and intestinal mucosal layer (ML) were observed in Bio−Se group compared to the control group (Figure 4A,B), which was 1.2-fold (*p* < 0.05) and 1.2-fold (*p* > 0.05) of that in the control group, respectively (Figure 4C,D).

### 2.5. Intestinal Flora and OTUs

**Figure 5:**
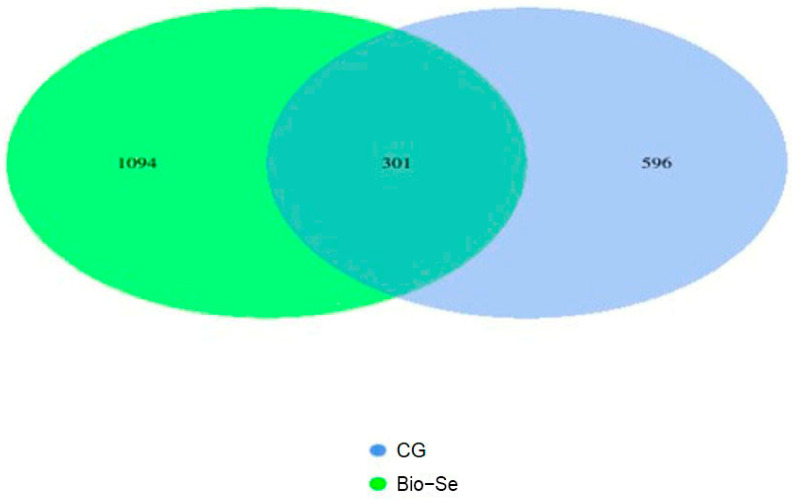
The Venn diagram comparing the OTUs in *E. sinensis* between the control group and the Bio−Se group. CG, control group (0 mg/kg of Bio−Se); Bio−Se, Bio−Se group (1.5 mg/kg of Bio−Se).

A total of 481,957 clean reads were obtained from the sequencing of 6 intestinal microbial samples, with an average of 80,326 reads per individual sample. The sequence lengths ranged from 423 to 427 base pairs. A Venn diagram analysis revealed that 301 operational taxonomic units (OTUs) were shared by the control and Bio−Se groups (Figure 4). Notably, an average of 1094 OTUs and 596 OTUs were unique in the Bio−Se group and control group, respectively (Figure 5).

### 2.6. Intestinal Microbial Diversity and Composition

The percentage coverage of each group exceeded 99.9%, and the majority of bacterial species present in the samples were identified. The results of observed features showed that more species were observed in the Bio−Se group. The results of dominance and pielou_e showed that the distribution of species observed in the control group (CG) was more uniform than that in the Bio−Se group (Bio−Se) (Table 2).

**Figure 6:**
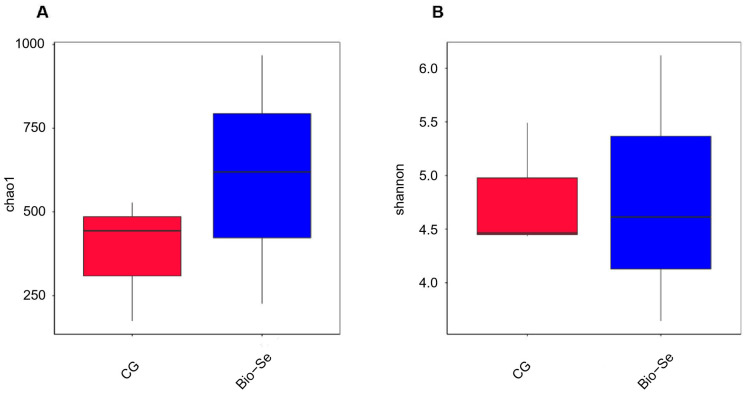
Alpha diversity analysis of the intestinal microbial communities of *E. sinensis* in the control group (CG) and Bio−Se group (Bio−Se) according to the (**A**) Chao-1 and (**B**) Shannon indexes. Chao-1 and Shannon show the total number of species and classifications in the community sample, respectively. CG, control group (0 mg/kg of Bio−Se); Bio−Se, Bio−Se group (1.5 mg/kg of Bio−Se).

**Figure 7:**
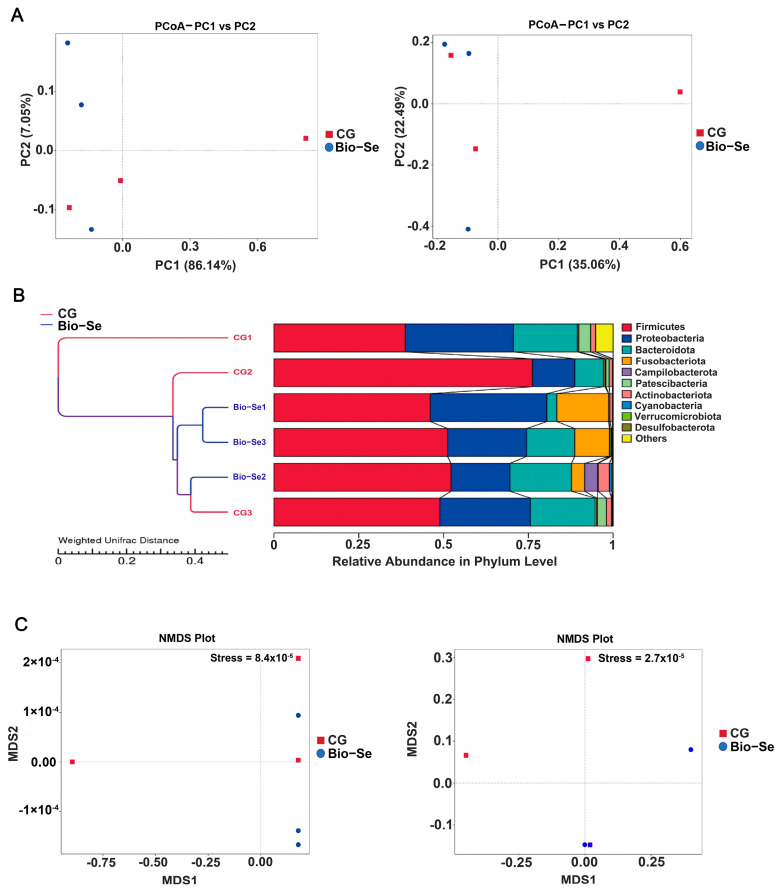
The beta diversity of intestinal microbiota of *E. sinensis* in the control group (CG) and Bio−Se group (Bio−Se) was evaluated via (**A**) PCoA, (**B**) UPGMA clustering of samples, and (**C**) NMDS. PCoA, principal coordinates analysis; UPGMA, unweighted pair-group method with arithmetic means; NMDS, non-metric multidimensional scaling; CG, control group (0 mg/kg of Bio−Se); Bio−Se, Bio−Se group (1.5 mg/kg of Bio−Se).

The Chao 1 index indicated that the Bio−Se group had a greater richness in intestinal microbial communities compared to the control group (Figure 6A). The Shannon Index was utilized to assess the species diversity of the intestinal microbiota; the results showed that intestinal microbial diversity was higher in the Bio−Se group (Figure 6B). The principal coordinate analysis (PCoA) of microbial communities revealed the separation between the control group and the Bio−Se group (Figure 7A). Meanwhile, two different separate classifications of bacterial communities were also observed between the control group and the Bio−Se group (Figure 7B). The non-metric multidimensional scaling (NMDS) analysis further confirmed the difference in microbial community structure between the two groups (Figure 7C).

**Figure 8:**
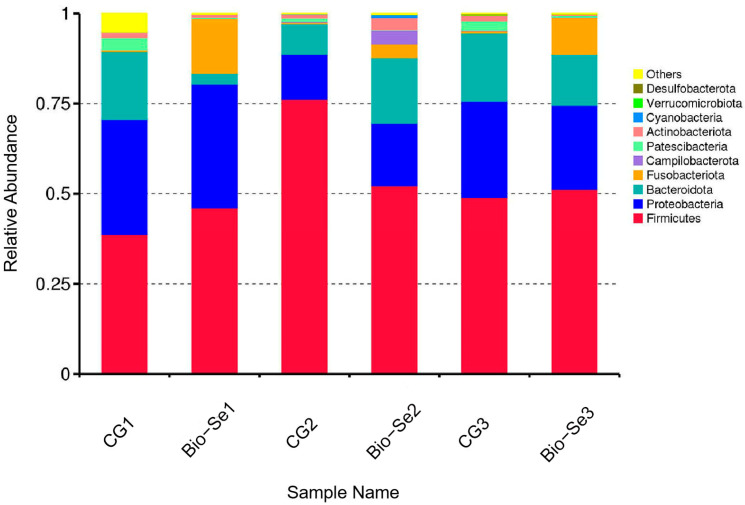
Effects of dietary Bio−Se on the relative abundance of intestine microbiota in *E. sinensis* at the phylum level (*n* = 3). CG, control group (0 mg/kg of Bio−Se); Bio−Se, Bio−Se group (1.5 mg/kg of Bio−Se).

The intestine microbial population composition at the phylum level is shown in Figure 7. The dominant intestine bacterial species were *Firmicutes* (54.62%), *Proteobacteria* (23.66%), and *Bacteroidetes* (15.47%), accounting for 93.75% of the total phylotypes (Figure 8, Table 3). Similarly, *Firmicutes* (49.81%), *Proteobacteria* (25.02%), and *Bacteroidota* (11.77%) were also the top three phyla in the Bio−Se group, accounting for 86.60% of all phylotypes (Figure 8, Table 3). Moreover, the *Fusobacteriota* abundance was increased, and in the Bio−Se group, the relative abundance of *Fusobacteriota* was about 24.5-fold that in the control group (Figure 8, Table 3).

**Figure 9:**
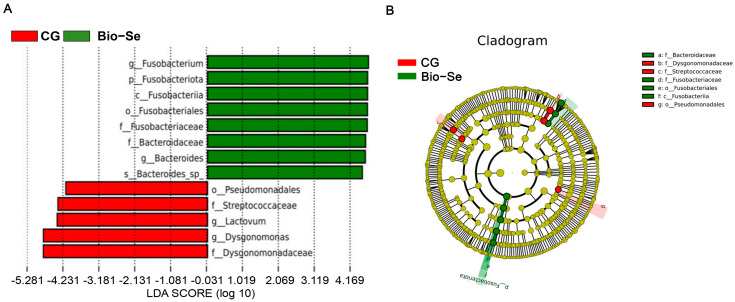
LefSe analysis of intestinal microbes in control group (CG) and Bio−Se group (Bio−Se) of *E. sinensis*. (**A**) Linear discriminate analysis (LDA) value distribution histogram; (**B**) cladogram from linear discriminant analysis effect size (LefSe) analysis. CG, control group (0 mg/kg of Bio−Se); Bio−Se, Bio−Se group (1.5 mg/kg of Bio−Se).

Furthermore, the LEfSe analysis was applied to identify the variation of intestinal microbes after Bio−Se feeding (Figure 9). There were seven bacterial taxa, including *Bacteroides*, *Bacteroidaceae*, *Fusobacteriaceae*, *Fusobacteriales*, *Fusobacteriia*, *Fusobacteriota*, and *Fusobacterium,* that were enriched in the Bio−Se group. Meanwhile, *Dysgonomonadaceae*, *Dysgonomonas*, *Lactovum*, *Streotooccaceae*, and *Pseudomonadale* were enriched in the control (CG) group.

## 3. Discussion

It has been demonstrated that an appropriate amount of Se is beneficial to the organism, and Se deficiency has a negative effect on aquatic animal health [42,43]. In this study, compared with other groups, feeding with 0.6 and 1.5 mg/kg of Bio−Se significantly enhanced the WGR and SGR in *E. sinensis*, while the WGR was inhibited in the crabs fed with 3.0 mg/kg Bio−Se diets. Similarly, it has been shown that moderate levels of dietary Se can increase the weight gain rate, while both the lowest and maximum levels of Se reduced weight gain in *M. nipponense* [9]. At present, several investigations have indicated that the connection between Se and growth performance was mostly attributed to the control of thyroxine production by selenium-containing deiodinase in mammals [44,45]. As a Se-containing amino acid, thyroxine plays a positive role in promoting the metabolism of protein, lipid, sugar and salt of the organism, thus increasing the growth and development of animals [46]. In addition, it was also found that the excessive dietary Se suppressed the growth of aquatic animals. Excessive Bio−Se can significantly decrease the growth rate and protein efficiency of sea cucumbers [23]. An excessive concentration of Se in the diet of *E. sinensis* leads to a decrease in weight gain rate [41]. The high Se levels caused disruptions in metabolic processes related to the balance of nutrients and energy, thus ultimately causing negative effects on the growth of *H. discus* [47].

In addition to promoting growth, Se supplementation has also been proposed to enhance antioxidation status and immunity [41]. In invertebrates, the antioxidant defense is essential for the immune system [48,49]. The SOD and GPX can promote antioxidant activity by reducing reactive oxygen species [50,51]. Moreover, as a Se-enriched enzyme, GPX exhibits an effective antioxidant capacity [52,53]. In the present research, with the increase in the dietary Bio−Se concentration (0, 0.3, 0.6, and 1.5 mg/Kg Bio−Se), the SOD and GPX activities also increased, and the highest activities were observed in the 1.5 mg/kg Bio−Se group. Similarly, it has also been reported that the appropriate concentration of Se (1.0 mg/kg) markedly elevated the activities of SOD and GPX in the serum of *Oreochromis niloticus* [8] and in *L. vannamei* [39]. In organisms, MDA serves as a marker for assessing oxidative stress-induced cellular damage [54]. It has been reported that the lipid peroxidation degree was reduced, and the MDA level was elevated in the shrimps fed insufficient Se [39]. Moreover, research has also revealed that Se deficiency results in high MDA toxicity, which finally damages the hepatopancreas cells of crabs [28]. In this study, the MDA concentration was decreased in the 0.6 mg/kg Bio−Se and 1.5 mg/kg Bio−Se groups while significantly up-regulated in the 3.0 mg/kg Bio−Se group. Our results collectively indicated that the appropriate concentration of dietary Bio−Se can enhance the resistance to oxidative stress in *E. sinensis*.

AMPs are the key components of the innate immune system in crustaceans [55,56]. The ALFs, Crus, and LYS are the AMPs with broad-spectrum antimicrobial activity [57,58,59]. In the present research, the expression levels of ALF-1, Crus-1, and LYS were all highly expressed in the 1.5 mg/kg Bio−Se group, while they were the least expressed in the 3.0 mg/kg Bio−Se group, which indicated that moderate amounts of Bio−Se might promote AMPs expression, while excessive Bio−Se had an inhibitory effect. Furthermore, it has been confirmed that the extracellular signal-regulated kinase (ERK) and Relish play vital roles in regulating the expression levels of AMPs expression [60,61]. ERK is the key element in the mitogen-activated protein kinase pathway [61], while Relish serves as a pivotal element within the immune deficiency (IMD) signaling pathway [60]. Although certain investigations have demonstrated that Se can regulate apoptosis via JNK, ERK, and p38 pathways [62], the regulatory function of Se on the expression of ERK and Relish was poorly understood in crustaceans. In this research, the results revealed a significant increase in the expression level of ERK in the 1.5 mg/kg Bio−Se group, whereas the lowest expression was observed in the 3.0 mg/kg Bio−Se group. The expression level of Relish was highly expressed in the 0.3 and 1.5 mg/kg Bio−Se group, while it was the least expressed in the 3.0 mg/kg Bio−Se group. Previous studies have indicated that Se can enhance oxidative stress resistance and innate immune function by regulating immune signaling pathways. Se can activate the ERK/p38 MAPK (mitogen-activated protein kinase) signaling pathway to protect against hydrogen peroxide-induced cell damage in porcine intestinal epithelial cells [63]. In addition, the methionine Se has been found to counteract the MAPK pathway, thereby preventing necroptosis occurrence in chickens [64]. Taken together, these results indicate that moderate amounts of Bio−Se can enhance the expression levels of AMPs by facilitating ERK and IMD signaling pathways.

Furthermore, recent studies have demonstrated that the morphology and microbial diversity of the intestine were essential for the immune system of crustaceans [65,66,67,68]. To assess the impact of Bio−Se on the immune system of crustaceans, the intestinal morphological and intestinal microbial diversity were investigated in the 1.5 mg/kg Bio−Se group. Plica thickness and mucosal layer thickness are important indicators of the intestinal morphological characteristics of crustaceans [69]. The intestinal mucosal layer can facilitate the process of osmosis and absorption of nutrients, as well as defend against intestinal microorganisms and inflammatory factors [70,71]. In this study, the thickness of intestinal plica and intestinal mucosal layer significantly increased after 1.5 mg/kg of Bio−Se supplementation. Similarly, it has been demonstrated that nanoparticles Se increased the thickness of the intestinal mucosal layer in *Carassius auratus*, suggesting that it could enhance intestinal immunity [72]. Additionally, Se can increase the villus height and villus width of juvenile fish and reduce the damage of high-fat diet on the intestinal tract, finally to maintain intestinal integrity [73]. In mammals, it has also been found that dietary Se can significantly enhance digestion and absorption by increasing the height of intestinal folds and the surface area of the intestines [71].

Multiple studies have shown that the intestinal microbiota plays a crucial role in preserving intestinal balance and enhancing the integrity and immunity of intestinal mucosal [74]. The healthy and balance intestinal microbiota promoted immunity by producing beneficial metabolites [75,76]. In this study, the 16S rDNA of the intestinal microbiota of *E. sinensis* was sequenced using second-generation sequencing technology. The results showed that the numbers of intestinal flora in 1.5 mg/kg Bio−Se group were significantly higher than that of control group. Then, further studies showed that the intestinal microbiota diversity index including Chao1 and Shannon were significantly (*p* < 0.05) increased after feeding with 1.5 mg/kg dietary Bio−Se. Previous studies have demonstrated that Se was beneficial for maintaining gut microbiota richness and diversity [77]. Similarly, the alpha diversity of gut microbiota was up-regulated in the dietary nano-Se supplementation group in *Ctenopharyngodon idella* [73]. Se was found to have a positive effect on alleviating the inflammation of the carp by altering the intestinal microbial composition [77].

Generally, *Firmicutes*, *Bacteroidetes*, and *Proteobacteria* are the predominant phyla of intestinal microbiota in *E. sinensis*, which play important roles in the digestive processes and maintaining intestinal health [78,79,80]. In this study, the numbers of dominant bacteria of intestinal flora were changed, while the types of dominant bacteria were not changed at the phylum level after being fed with 1.5 mg/kg of dietary Bio−Se. The dominant flora in both the Bio−Se group and control group were *Firmicutes*, *Bacteroidetes*, and *Proteobacteria*, which was consistent with previous research results. It is worth mentioning that the number of *Fusobacteria* in the 1.5 mg/kg Bio−Se group was significantly higher compared to the control group, indicating the positive impact of the Bio−Se supplementation on intestinal health in *E. sinensis*. *Fusobacteria* is butyrate-producing anaerobic bacteria that can ferment amino acids and carbohydrates [81]. It has been documented that *Fusobacteria* possesses immunomodulatory and anti-inflammatory properties by producing butyric acid in the intestine [82]. Moreover, it has also been reported that dietary nano-Se effectively normalized the intestinal microbiota imbalance in *C. idella* by increasing the abundance of beneficial bacteria, such as *Fusobacteria* [73].

## 4. Materials and Methods

### 4.1. Diets Preparation

For the preparation of dietary Bio−Se fed, 10 kg of wheat bran, 10 kg of sterile water (heat sterilization 120 °C, 20 min), and 35 g of sodiumselenite were firstly mixed in a sterilized 250-L bioreactor. Then, the compound lactobacillus (Dalian Baiantai Biotechnology Co., Ltd., Dalian, China) with a concentration of 5 × 10^5^ CFU/mL was added. Finally, the mixture continued to be fermented at 30 °C for 5 days, which was produced as Bio−Se [23]. The Bio−Se was dried at 105 °C for 4 h and smashed by a pulverizer with 0.25 mm before using it. The concentrations of Se were measured, and the content of organic Se in the dried samples accounted for more than 98%, according to the report [23]. The Bio−Se was added into the basal diet to formulate different experimental diets. The final Bio−Se concentrations in the five diets were 0, 0.3, 0.6, 1.5, and 3.0 mg/kg diet. The proximate composition of the basal diet is shown in Table 4.

### 4.2. Experimental Animals and Management Procedure

The Chinese mitten crabs were purchased from Lianyungang, Jiangsu Province, China. Before experiment, crabs were fed with basal diet for one week under laboratory conditions. Subsequently, a total of 150 healthy crabs (about 16 g) were randomly allocated into 15 plastic tanks (80 × 100 cm), with 10 crabs per tank. Three were three replicates for per dietary group (0, 0.3, 0.6, 1.5, and 3.0 mg/kg Bio−Se group). Crabs were fed twice daily (09:00 and 16:00) at 3% of their body weight for four weeks. During the feeding trial, the water temperature was about 26 ± 1 °C, and the water was continuously aerated by air stones, with a daily water exchange rate of 1/2 of the tank volume. The Se concentration in the water was lower than 0.02 mg/L. All crab experiments were performed in accordance with the approval and guidelines of the Animal Ethics Committee of Dalian Ocean University (Permit Number: DLOU2023008).

### 4.3. Sample Collection

After 4 weeks of feeding, five crabs were randomly selected from each tank to dissect the hepatopancreas and intestine. Then, the hepatopancreas were stored at −80 °C to use for the detection of enzymatic activity and gene expression. The intestines were collected for the section staining and intestinal flora determination.

### 4.4. Growth Parameters Analysis

The initial and final weight of crabs in each group was recorded. Growth performance, including weight gain rate (WGR), specific growth rate (SGR), and survival rate (SR), was measured using the following parameters:

WGR (%) = 100 × (final weight − initial weight)/initial weight;

SGR (%/day) = 100 × (ln final body weight − ln initial body weight)/breeding days;

SR (%) = 100 × (final number of survival crabs/initial number of crabs).

### 4.5. Biochemical Analysis

The frozen hepatopancreas were homogenized in normal saline to obtain hepatopancreas crude extract for the measurement of enzymatic activity. The activities of antioxidant enzymes (ACP, SOD, MDA, and GPX) were quantified by commercially available kits (Jiancheng, Nanjing, China), according to the guidelines provided by the manufacturer with some modifications, respectively [83,84,85]. Briefly, the hepatopancreas crude extract was combined with the colorimetric substrate and allowed to incubate at 37 °C. After adding the stop solution, the absorbance was recorded by the precision microplate reader (BioTek, Winooski, VT, USA).

### 4.6. Quantitative Real-Time PCR Analysis (qRT-PCR)

The total RNA was isolated from the hepatopancreas using the Trizol reagent (Invitrogen, Carlsbad, CA, USA). Subsequently, the cDNA was synthesized with 1 μg of total RNA using Prime Script™ RT reagent Kit with gDNA Eraser (Takara, Otsu, Shiga, Japan). The qRT-PCR reaction mixture was prepared with the SYBR^®^ Premix Ex Taq™ (Takara, Japan) and the PCR amplification by the ABI PRISM 7500 Sequence Detection System. The gene-specific primers of *Es*ERK (GenBank accession No. KP100030.1), *Es*Relish (GenBank accession No. GQ871279.1), *Es*ALF-1 (GenBank accession No. OR813948.1), *Es*Crus-1 (GenBank accession No. FJ974138.1), *Es*LYS (GenBank accession No. JN416111.1), and *Es*β-actin (GenBank accession No. HM053699) are shown in Table 5. *Es*β-actin was used as the internal control. The relative mRNA expression levels were determined using the 2^−∆∆Ct^ method [86].

### 4.7. Analysis of Intestinal Morphology

The crab intestines were fixed in a 4% polyformaldehyde solution for 24 h. Then, the samples were dehydrated in ethanol and were finally embedded in paraffin wax. The intestine sections were stained using hematoxylin and eosin (HE) [87]. The intestine plica thickness and mucosal layer thickness were observed by Eclipse Ci-L photographic microscope and quantified by employing Image-Pro Plus 6.0 analysis software.

### 4.8. Analysis of Bacterial Diversity in the Intestine

The genomic DNA from bacterial intestinal samples was extracted utilizing the TIANamp Stool DNA Kit (TIANGEN, Beijing, China). The purity and DNA concentration were assessed using the NanoDrop 2000 (Thermo Scientific, Wilmington, DE, USA. The hypervariable V3–V4 regions of the bacterial 16S rRNA gene were targeted for amplification using universal primers (Table 5) via PCR system [88]. Then, the purified PCR products were paired-end sequenced on an Illumina MiSeq platform (Illumina, San Diego, CA, USA) by Novogene Co., Ltd. (Beijing, China). The raw sequencing data were paired using Fast Length Adjustment of Short Reads (FLASH) [89]. The operational taxonomic units (OTUs) were generated by the qualified reads utilizing the Quantitative Insights into Microbial Ecology (QIIME) software version 1.8.0 [90], and the chimeric sequences were identified using the UCHIME algorithm [91].

The alpha diversity indexes, including Chao1 and Shannon, were analyzed by comparing the control group and Bio−Se group. For the beta diversity, the principal coordinate analysis (PCoA) was performed based on UniFrac metrics. The intestinal microbial composition between the control group and Bio−Se group was identified at the phylum and genus levels. Linear discriminant analysis (LDA) effect size (LEfSe) analysis was performed to identify differential bacterial taxa.

### 4.9. Statistical Analysis

The data were analyzed using SPSS version 26.0 software and were graphed using GraphPad Prism 8.0. Statistical significance was calculated by one-way ANOVA. If overall differences were significant, the Turkey multiple comparison test was also used among different groups.

## 5. Conclusions

In summary, the present results indicated that appropriate Bio−Se dietary consumption could improve the growth, anti-oxidative potential, immune function, intestinal morphology, and intestinal flora richness of *E. sinensis*. The WGR, SGR, and SR were highest in the 1.5 mg/kg Bio−Se feeding group. The activities of antioxidant enzymes, including ACP, SOD, and GPX in hepatopancreas, increased with increasing dietary Bio−Se dosage (0, 0.3, 0.6, and 1.5 mg/kg of Bio−Se) and peaked in the 1.5 mg/kg Bio−Se group. Similarly, the expression levels of antimicrobial peptide genes (ALF-1, Crus-1, and LYS) and key molecules of the immune-signaling pathway (ERK and Relish) were also up-regulated in the 1.5 mg/kg Bio−Se group and were decreased in the 3.0 mg/kg Bio−Se group. Together, our study showed that an appropriate amount of Bio−Se can improve intestinal morphology and increase intestinal plica and mucosal layer thickness, as well as the intestinal dominant flora richness of Chinese mitten crabs. Based on the broken-line model analysis of WGR for the dietary Bio−Se level, the optimum dietary Bio−Se was suggested to be 1.1 mg/kg. These results provide experimental evidence for the effects of Bio−Se on the growth and immunity of aquatic crustaceans, which may contribute to the application of Bio−Se in aquaculture farming.

## Figures and Tables

**Table 1:** Growth performance of *E. sinensis* fed with the experimental diets containing different Bio−Se concentrations.

Bio−Se (mg/kg)	W_t_ (g)	WGR (%)	SGR (%/day)	SR (%)
0	18.14 ± 0.34 ^c^	11.81 ± 0.27 ^d^	0.38 ± 0.03 ^bc^	63.33 ± 6.67 ^b^
0.3	18.47 ± 0.32 ^b^	12.54 ± 0.41 ^c^	0.42 ± 0.03 ^bc^	85.93 ± 2.97 ^a^
0.6	18.70 ± 0.49 ^ab^	13.71 ± 0.48 ^b^	0.45 ± 0.03 ^ab^	80.96 ± 0.94 ^a^
1.5	18.87 ± 0.45 ^a^	14.74 ± 0.48 ^a^	0.50 ± 0.03 ^a^	87.43 ± 1.73 ^a^
3.0	18.11 ± 0.32 ^c^	10.32 ± 0.13 ^d^	0.36 ± 0.04 ^c^	69.83 ± 0.17 ^b^

W_t_, final individual weight; WGR, weight gain rate; SGR, specific growth rate; SR, survival rate. The data are shown as mean ± standard deviation. Values in the same column with the same letter indicated no significant difference (*p* > 0.05), while those with different letters indicate significant differences compared to other groups (*p* < 0.05).

**Table 2:** Intestine microbe diversity of *E. sinensis* in control group (CG) and Bio−Se group (Bio−Se).

Group	Chao1	Observed_Features	Pielou_e	Shannon	Simple	Coverage
CG	362.04 ± 27.95 ^a^	317.06 ± 51.27 ^a^	0.63 ± 0.12 ^a^	4.78 ± 0.00 ^a^	0.91 ± 0.02 ^a^	0.999 ± 0.00 ^a^
Bio−Se	552.06 ± 62.94 ^b^	438.17 ± 94.29 ^b^	0.59 ± 0.13 ^a^	4.77 ± 0.02 ^a^	0.90 ±0.02 ^a^	0.997 ± 0.01 ^a^

CG, control group (0 mg/kg of Bio−Se); Bio−Se, Bio−Se group (1.5 mg/kg of Bio−Se). Data are shown as means ± SD (*n* = 3). Values in the same column with different lettered superscripts indicate significant difference (*p* < 0.05), and those with the same letters indicate no significant difference (*p* > 0.05).

**Table 3:** The proportion (%) of the number of intestinal bacteria in *E. sinensis* in control group (CG) and Bio−Se group (Bio−Se).

Taxonomy	*Firmicutes*	*Proteobacteria*	*Bacteroidota*	*Fusobacteriota*	*Campilobacterota*
CG	54.62	23.66	15.47	0.45	0.16
Bio−Se	49.81	25.02	11.77	9.80	1.32

CG, control group (0 mg/kg of Bio−Se); Bio−Se, Bio−Se group (1.5 mg/kg of Bio−Se).

**Table 4:** Ingredients and nutrient composition of the basic diet (% dry weight).

Ingredient	(%)
Fish meal ^1^	35.0
Bean meal	25.0
Peanut meal	16.0
Corn starch	5.7
Soybean oil ^2^	4.0
yeast	5.0
Cellulose	4.7
Vitamin mix ^3^	2.0
Se-free mineral mix ^4^	2.0
Cholesterol	0.3
Choline chloride	0.2
Ethopabate	0.01
Calcium propionate	0.10
Crude protein	44.8
Crude lipid	7.8

^1^ Fish meal: crude protein 68.1% dry matter, crude lipid 10.2% dry matter, Qingdao Qihao Biotechnology Company (Qingdao, Shandong Province, China). ^2^ Soybean meal: crude protein 43.4% dry matter, crude lipid 1.9% dry matter, Qingdao Qihao Biotechnology Company (Qingdao, Shandong Province, China). ^3^ Vitamin mix (mg or g kg^−1^ diet): vitamin D, 5 mg; vitamin K, 10 mg; vitamin B12, 10 mg; vitamin B6, 20 mg; folic acid, 20 mg; vitamin B1, 25 mg; vitamin A, 32 mg; vitamin B2, 45 mg; pantothenic acid, 60 mg; biotin, 60 mg; niacin acid, 200 mg; a-tocopherol, 240 mg; inositol, 800 mg; ascorbic acid, 2000 mg; microcrystalline cellulose, 16.47 g. ^4^ Se-free mineral mix (g kg^−1^ diet): KCl, 0.84 g; MgSO_4_·7H_2_O, 3 g; NaH_2_PO_4_, 6.45 g; KH_2_PO_4_, 3 g; Ca(H_2_PO_4_)_2_·H_2_O, 7.95 g; CaCO_3_, 3.15 g; C_6_H_10_CaO_6_·5H_2_O, 4.95 g; FeC_6_H_5_O_7_·5H_2_O, 0.36 g; CuSO_4_·5H_2_O, 0.1055 g; ZnSO_4_·7H_2_O, 0.1428 g; MnSO_4_·H_2_O, 0.0321 g; AlCl_3_·6H_2_O, 0.0045 g; CoCl_2_·6H_2_O, 0.042 g; KI, 0.0069 g.

**Table 5:** Sequences of the primers used in the study.

Primer	Sequence (5′-3′)
*Es*ERK-F	TTCAGCAACAGGCTCATC
*Es*ERK-R	TGTTCAGGAGGAGGTTTGATGGC
*Es*Relish-F	TCTCCCTACTCTGACCATTCC
*Es*Relish-R	TTCCCACCATCTCACTCTTGT
*Es*ALF-1-F	GACGCAGGAGGATGCTAAC
*Es*ALF-1-R	TGATGGCAGATGAAGGACAC
*Es*Crus-1-F	GCTCTATGGCGGAGGATGTCA
*Es*Crus-1-R	CGGGCTTCAGACCCACTTTAC
*Es*LYS-F	CTGGGATGATGTGGAGAAGTGC
*Es*LYS-R	TTATTCGGTGTGTTATGAGGGGT
*Esβ-actin*-F	GCATCCACGAGACCACTTACA
*Esβ-actin*-R	CTCCTGCTTGCTGATCCACATC
*Es*V3-V4-F *Es*V3-V4-R	CCTACGGGAGGCAGCAG GACTACCAGGGTATCTAATC

## Data Availability

Data are contained within the article. All the data can be provided by the corresponding author upon request.
